# Machine Learning-Based Prediction of Food Allergy in Children Aged <2 Years with Atopic Dermatitis

**DOI:** 10.3390/jcm15155941

**Published:** 2026-07-30

**Authors:** Enes Çelik, Ahmed Cihad Genç, Hande Yüksel Bulut, Mehmet Akif Kaya, Fatma Dindar Çelik

**Affiliations:** 1 Division of Pediatric Allergy and Immunology, Department of Pediatrics, Ankara Atatürk Sanatorium Training and Research Hospital, Ankara 06290, Türkiye; handeyuksel_88@hotmail.com; 2 Private Clinic, İstanbul 34480, Türkiye; genccihad@gmail.com; 3 Division of Pediatric Allergy and Immunology, Department of Pediatrics, Alanya Alaaddin Keykubat University, Antalya 07425, Türkiye; akifkaya@gmail.com; 4 Department of Immunology and Allergy, Ankara Atatürk Sanatorium Training and Research Hospital, Ankara 06290, Türkiye; fatmadindar@gmail.com

**Keywords:** artificial intelligence, atopic dermatitis, food allergy, infant, machine learning, prediction

## Abstract

**Background/Objectives**: Children with atopic dermatitis (AD) are at increased risk of food allergy (FA). This study aimed to develop and evaluate machine learning (ML) models for predicting FA in children aged <2 years with AD and to assess feature contribution. **Methods**: This retrospective study included children aged 1 month to 2 years who were diagnosed with AD and underwent evaluation for FA. Demographic characteristics, clinical features, and laboratory findings were extracted from electronic medical records. Four ML algorithms—CatBoost, XGBoost, LightGBM, and logistic regression—were evaluated for predicting FA. **Results**: A total of 435 children with AD were included, of whom 185 (42.5%) were female. The median age at presentation was 7.4 months (IQR, 4.8–10.8), and FA was present in 101 patients (23.2%). Among all evaluated models, CatBoost using clinical features alone demonstrated the best overall performance, achieving an AUC of 0.91 (95% CI, 0.84–0.97), with a sensitivity of 0.85 and a specificity of 0.91. Using combined clinical and laboratory features, XGBoost achieved an AUC of 0.88 (95% CI, 0.79–0.95), with a sensitivity of 0.70 and a specificity of 0.96. Using clinical features alone, LightGBM and logistic regression achieved AUCs of 0.89 (95% CI, 0.82–0.96) and 0.86 (95% CI, 0.75–0.95), respectively, with sensitivities of 0.85 and 0.65 and specificities of 0.82 and 0.88. **Conclusions**: CatBoost demonstrated the best overall performance in predicting FA in children aged <2 years with AD using clinical features alone. ML models may help identify children with AD who require further diagnostic evaluation for FA.

## 1. Introduction

Atopic dermatitis (AD) is the most prevalent chronic inflammatory skin disorder worldwide, commonly manifesting within the first year of life and affecting up to 15–25% of children. It is a multifactorial condition resulting from a complex interplay between genetic predisposition, immune dysregulation, and environmental factors [1].

Food allergy (FA) results from exposure to specific food protein antigens and may present with urticaria, angioedema, gastrointestinal symptoms, respiratory involvement, or anaphylaxis [2]. In individuals with concomitant AD, eczematous lesions may worsen within hours to days following food exposure, whereas elimination of the offending foods may lead to improvement or even prevention of disease manifestations [3].

Impairment of the skin barrier is recognized as a pivotal initial step in the process of epicutaneous sensitization to food allergens, ultimately contributing to the development of FA [4,5]. This association is further supported by evidence demonstrating an increased risk of FA in patients with AD [6,7]. Approximately one-third of children with AD develop FA [3]. More severe and persistent forms of AD are more commonly linked to FA [6].

Artificial intelligence (AI), a subfield of computer science, is increasingly recognized for its potential to transform medicine by simulating human cognitive functions and performing tasks that traditionally require human intelligence [8]. Building on these advances, machine learning (ML) has emerged as a core component of AI, enabling algorithms to learn from large, structured clinical datasets and thereby enhance diagnostic accuracy [9,10]. In allergy and immunology, AI has demonstrated a wide range of applications, including the prediction of asthma through analysis of electronic health record data, the diagnosis of allergic diseases using epigenetic profiles, and the identification of eczema from image-based datasets [9]. In pediatric patients with AD, studies investigating the use of ML to predict FA remain limited in the current literature.

The aim of this study was to develop and evaluate ML models for predicting FA in children under 2 years of age with AD and to determine the relative importance of clinical and basic laboratory features contributing to model performance.

## 2. Materials and Methods

### 2.1. Study Design and Population

**Figure 1:**
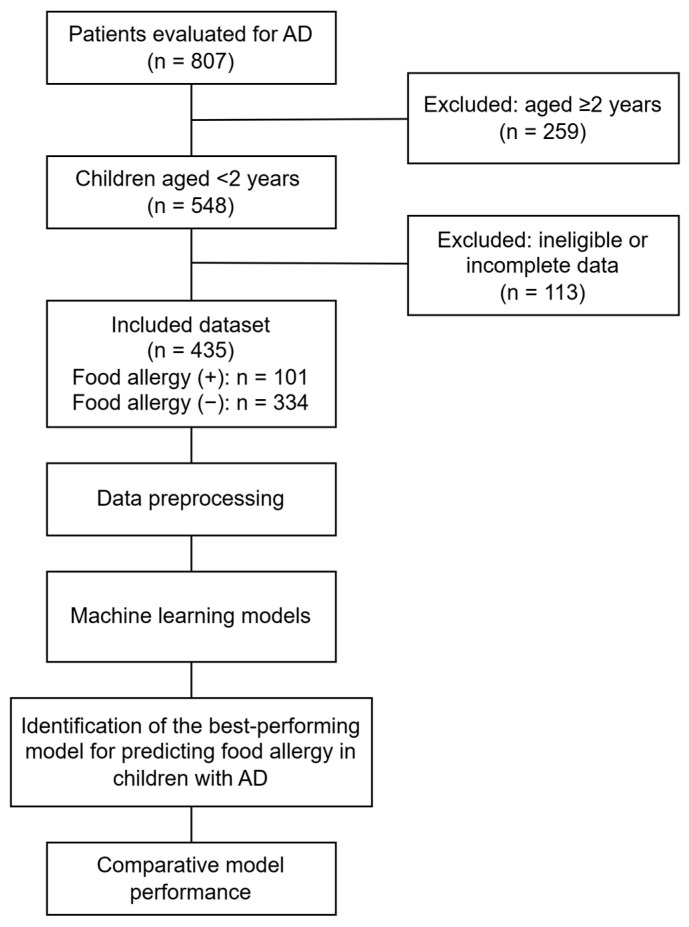
Flowchart of patient selection and machine learning analysis for predicting food allergy in children aged <2 years with atopic dermatitis. AD, atopic dermatitis.

This retrospective study was conducted at the Division of Pediatric Allergy and Immunology, Ankara Atatürk Sanatorium Training and Research Hospital, Ankara, Türkiye, between 1 June 2023 and 31 December 2025. Children aged 1 month to 2 years who were diagnosed with AD and underwent evaluation for FA were included. The diagnosis of AD was established according to the Hanifin and Rajka criteria [11]. Patients with underlying chronic conditions such as immunodeficiency, malignancy, or metabolic disorders, as well as those who were not assessed for FA or had incomplete data, were not eligible for inclusion. The patient selection process and ML analysis workflow are summarized in Figure 1. Demographic characteristics, clinical features, and laboratory findings of the patients were extracted from electronic medical records.

### 2.2. Clinical Scores

Disease severity was assessed using the SCORAD (Scoring Atopic Dermatitis) index at the initial presentation [12]. Based on SCORAD values, AD was classified as mild (<25), moderate (25–50), or severe (≥50). For ML analyses, SCORAD was entered as a categorical variable (mild, moderate, and severe).

The distribution of eczema was further evaluated using the Sites of Eczema (SoE) scores, which assess four anatomical regions (head/neck, trunk, upper extremities, and lower extremities), with each region assigned 1 point if eczema was present, yielding a maximum score of 4 [13].

### 2.3. Assessment of Food Allergy

FA was assessed using clinical history, evidence of allergic sensitization, and oral food challenge (OFC) findings, as appropriate. All patients underwent skin prick testing (SPT) and/or serum-specific IgE (sIgE) measurements. SPTs were conducted using commercial extracts (Lofarma, Milan, Italy) with a panel including cow’s milk, hen’s egg, wheat, soy, peanut, sesame, hazelnut, and walnut. In addition, the test panel was individualized by incorporating specific foods suspected based on the patient’s clinical history. Histamine (10 mg/mL) and saline served as the positive and negative controls, respectively. A wheal diameter of at least 3 mm greater than that of the negative control on SPT was defined as positive. Total and sIgE levels were measured using a chemiluminescent immunoassay (IMMULITE 2000 XPI, Siemens, Erlangen, Germany). A sIgE level of ≥0.35 kU/L was considered indicative of sensitization.

Clinical reactivity was determined based on OFC findings when available, or on a convincing history of reproducible reactions together with documented sensitization to the suspected food. Open OFC was performed at least 15 days after resolution of the index AD flare and any FA-related symptoms. A challenge (or natural exposure) was regarded as positive when it elicited either: (i) at least one immediate symptom within 2 h of exposure—cutaneous (urticaria, angioedema), gastrointestinal (vomiting, diarrhea, abdominal pain), and/or respiratory (e.g., wheezing) manifestations, or anaphylaxis; or (ii) a food-triggered exacerbation of AD, defined as a ≥10-point increase in the SCORAD index from baseline, confirmed by physician assessment and followed by improvement after elimination of the implicated food [14,15,16,17].

### 2.4. Data Preprocessing

The final dataset comprised 435 children aged <2 years with AD who were included for the prediction of FA. The dataset was randomly partitioned into a training set comprising 80% of the patients (n = 348) and a holdout test set comprising the remaining 20% (n = 87). The training set was used throughout the model development process, including feature selection and hyperparameter tuning. The holdout test set was kept separate from model development and was used only for the final evaluation and reporting of model performance.

### 2.5. Modeling, Software and Libraries

**Figure 2:**
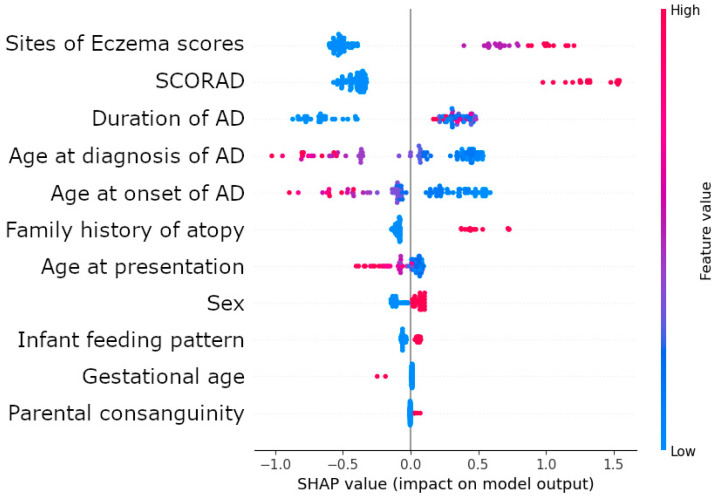
SHAP feature importance plot for the CatBoost model based on clinical features for predicting food allergy. AD, atopic dermatitis; SHAP, SHapley Additive exPlanations; SCORAD, SCORing Atopic Dermatitis.

All ML analyses were performed using Python 3.14. ML models were implemented with CatBoost, XGBoost, LightGBM, and scikit-learn (Logistic Regression). Hyperparameter optimization was conducted via RandomizedSearchCV with stratified 5-fold cross-validation. Feature importance was assessed using SHAP (SHapley Additive exPlanations) TreeExplainer for tree-based models (Figure 2) and permutation importance for Logistic Regression. Data manipulation and preprocessing were carried out using pandas and NumPy. A fixed classification threshold of 0.5 was applied to all models, and the independent test set was used solely for performance evaluation. Model performance metrics, including area under the curve (AUC), sensitivity, specificity, F1-score, and average precision, were computed using scikit-learn’s metrics module. Bootstrap confidence intervals (1000 resamples, 95% CI) were calculated for all test-set metrics. Visualizations, including receiver operating characteristic (ROC) curves, precision-recall curves, and confusion matrices, were generated using Matplotlib 3.10, Seaborn 0.13, and the SHAP library. Performance metric plots were generated using GraphPad Prism 11.0.2. All experiments were conducted with a fixed random seed (seed = 3) to ensure reproducibility.

### 2.6. Selected Features for ML Models

Four ML algorithms, namely CatBoost, XGBoost, LightGBM, and logistic regression, were evaluated for the prediction of FA. Each algorithm was assessed using two prespecified input feature sets. The clinical feature set included age at presentation, age at onset of AD, age at diagnosis of AD, duration of AD, sex, gestational age, infant feeding pattern, family history of atopy, parental consanguinity, SoE scores, and SCORAD index. The second feature set comprised the clinical features together with laboratory variables, including absolute eosinophil count, eosinophil percentage, and total IgE level. Thus, a total of eight model–feature set combinations were evaluated. The laboratory feature set was intentionally restricted to routinely available basic laboratory variables because the study aimed to evaluate whether FA risk could be estimated without incorporating allergen-specific diagnostic test results. Accordingly, food-specific IgE levels and skin prick test wheal sizes were not included in the prediction models.

### 2.7. Statistical Analysis

Statistical analyses were performed using IBM SPSS Statistics for Windows, version 22.0 (IBM Corp., Armonk, NY, USA). The normality of continuous variables was assessed using the Kolmogorov–Smirnov and Shapiro–Wilk tests. As all continuous variables showed non-normal distributions, they were presented as medians with interquartile ranges (IQR). Comparisons of continuous variables between two independent groups were performed using the Mann–Whitney U test. Categorical variables were summarized as numbers and percentages and compared using the chi-square test. A two-sided *p*-value of < 0.05 was considered statistically significant.

### 2.8. Ethical Approval

The study was conducted in accordance with the principles of the Declaration of Helsinki and received approval from the Scientific Research Ethics Committee of Ankara Atatürk Sanatorium Training and Research Hospital (Approval No: 2024-BÇEK/489). Because of the retrospective design of the study and the use of anonymized patient data, the requirement for written informed consent was waived by the ethics committee.

## 3. Results

The demographic and clinical characteristics of the study population according to FA status are presented in Table 1. A total of 435 children with AD were included in the study, of whom 185 (42.5%) were female. FA was present in 101 patients (23.2%). Compared with children without FA, those with FA had a significantly younger age at presentation [median (IQR): 6.1 (4.1–8.6) vs. 7.9 (5.2–11.3) months, *p* < 0.001], an earlier age at onset of AD [2.0 (2.0–3.0) vs. 4.0 (3.0–6.0) months, *p* < 0.001], and an earlier age at diagnosis of AD [4.0 (3.0–5.0) vs. 6.0 (4.0–9.0) months, *p* < 0.001]. In contrast, the duration of AD was significantly longer in the FA group than in the group without FA [3.0 (2.0–5.0) vs. 2.0 (1.0–4.0) months, *p* = 0.005]. Exclusive breastfeeding was less frequent among children with FA than among those without FA [47 (46.5%) vs. 194 (58.1%), respectively, *p* = 0.041], whereas a family history of atopy was more common in the FA group [28 (27.7%) vs. 52 (15.6%), respectively, *p* = 0.006]. Children with FA had higher AD severity according to the SCORAD classification and higher SoE scores than those without FA (both *p* < 0.001).

Hen’s egg was the most frequently identified food allergen among children with FA (n = 87), followed by cow’s milk (n = 21), tree nuts (n = 16), sesame (n = 12), and peanut/legumes (n = 7). Thirty-three patients (32.7%) had multiple food allergies. Laboratory parameters were significantly higher in children with FA than in those without FA, including absolute eosinophil count [median (IQR): 500 (340–910) vs. 260 (160–360) cells/µL, *p* < 0.001], eosinophil percentage [4.8 (3.4–8.0) vs. 2.8 (1.6–3.7)%, *p* < 0.001], and total IgE level [30.8 (12.6–91.2) vs. 16.3 (6.1–36.4) kU/L, *p* < 0.001].

**Figure 3:**
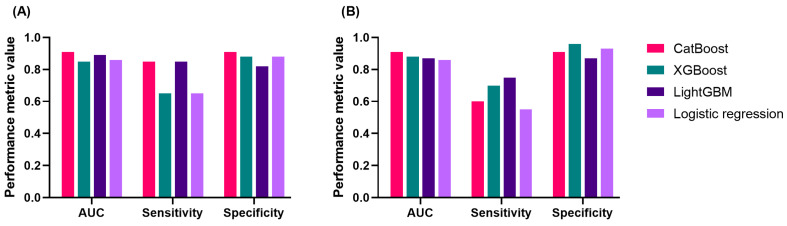
Performance metrics of the evaluated prediction models according to feature set. Area under the receiver operating characteristic curve (AUC), sensitivity, and specificity of CatBoost, XGBoost, LightGBM, and logistic regression models are presented using (**A**) clinical features alone and (**B**) combined clinical and laboratory features.

The performance of the CatBoost, XGBoost, LightGBM, and logistic regression models in predicting FA among children aged <2 years with AD is presented in Table 2. Final models were trained using two feature sets: clinical features alone and combined clinical and laboratory features. Model performance was subsequently evaluated on the holdout test dataset. Among the evaluated algorithms, CatBoost demonstrated the best overall predictive performance (Figure 3).

**Figure 4:**
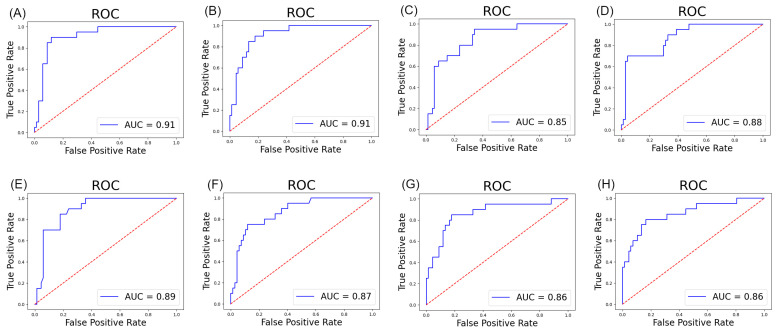
Receiver operating characteristic curves of the machine learning models for predicting food allergy in children aged <2 years with atopic dermatitis according to the included feature sets. (**A**) CatBoost model based on clinical features alone; (**B**) CatBoost model based on combined clinical and laboratory features; (**C**) XGBoost model based on clinical features alone; (**D**) XGBoost model based on combined clinical and laboratory features; (**E**) LightGBM model based on clinical features alone; (**F**) LightGBM model based on combined clinical and laboratory features; (**G**) logistic regression model based on clinical features alone; and (**H**) logistic regression model based on combined clinical and laboratory features. The red dashed diagonal line represents the no-discrimination line (AUC = 0.5). AUC, area under the curve; ROC, receiver operating characteristic.

The CatBoost model based on clinical features alone achieved an AUC of 0.91 (95% CI: 0.84–0.97), with a sensitivity of 0.85 and a specificity of 0.91. After the addition of laboratory features, including absolute eosinophil count, eosinophil percentage, and total IgE level, the AUC and specificity remained unchanged at 0.91 (95% CI: 0.84–0.97) and 0.91, respectively, whereas sensitivity decreased from 0.85 to 0.60. The corresponding ROC curves are shown in Figure 4A,B.

The XGBoost model based on clinical features alone achieved an AUC of 0.85 (95% CI: 0.76–0.93), with a sensitivity of 0.65 and a specificity of 0.88. After the addition of laboratory features, the AUC increased numerically to 0.88 (95% CI: 0.79–0.95), while sensitivity and specificity increased to 0.70 and 0.96, respectively. The corresponding ROC curves are shown in Figure 4C,D.

The LightGBM model based on clinical features alone achieved an AUC of 0.89 (95% CI: 0.82–0.96), with a sensitivity of 0.85 and a specificity of 0.82. After the addition of laboratory features, the AUC decreased numerically to 0.87 (95% CI: 0.78–0.94). Sensitivity decreased to 0.75, whereas specificity increased to 0.87. The corresponding ROC curves are shown in Figure 4E,F.

The logistic regression model based on clinical features alone achieved an AUC of 0.86 (95% CI: 0.75–0.95), with a sensitivity of 0.65 and a specificity of 0.88. After the addition of laboratory features, the AUC remained unchanged at 0.86 (95% CI: 0.75–0.95). Sensitivity decreased to 0.55, whereas specificity increased to 0.93. The corresponding ROC curves are shown in Figure 4G,H.

## 4. Discussion

In the present study, we investigated the performance of ML models in predicting FA among children aged <2 years with AD. To the best of our knowledge, this is the first study to evaluate ML-based FA prediction specifically in this population. All four evaluated models—CatBoost, XGBoost, LightGBM, and logistic regression—achieved AUC values of ≥0.85 on the holdout test dataset, indicating good discriminatory performance. Among these models, CatBoost demonstrated the best overall performance, achieving an AUC of 0.91, with a sensitivity of 0.85 and a specificity of 0.91, using clinical features alone.

AD is a major risk factor for FA in children, particularly during early childhood [15,18]. Previous studies have identified several characteristics associated with an increased likelihood of FA among children with AD. Younger age and greater AD severity have been associated with FA in this population [19]. Similarly, in a study involving infants aged <1 year with AD, Akelma et al. [13] reported that higher serum total IgE levels, increased eosinophil counts and percentages, greater SCORAD index values, and higher SoE scores were associated with FA. In the present study, we evaluated routinely collected demographic, clinical, and laboratory characteristics, several of which have previously been associated with FA in young children with AD. The inclusion of clinically relevant and readily obtainable predictors may have contributed to the strong discriminatory performance observed in our models.

Previous studies have evaluated predictive models for FA in broader pediatric populations. Landau et al. [20] used prenatal and early postnatal clinical variables available by 4 months of age, including demographic characteristics, familial atopic history, perinatal antibiotic exposure, and infant AD, to predict FA using a random forest model. Their model achieved an AUC of 0.80, with a sensitivity of 62% and a specificity of 84%. Notably, infant AD was included as a predictor in their general early-life model, whereas our study focused exclusively on children with established AD. More recently, Zhu et al. [21] developed a prediction model for IgE-mediated FA in children aged 0–3 years using early-life and familial factors, reporting an AUC of 0.778 in the overall dataset. Although AD was frequent among children with FA in their cohort, their model was not specifically developed for children with AD. Our study therefore provides additional evidence from a distinct high-risk population by evaluating FA prediction using clinical and laboratory characteristics and demonstrating that strong discriminatory performance can be achieved using clinical features alone.

Gryak et al. [22] developed ensemble ML models to predict peanut oral food challenge outcomes in children using clinical and laboratory variables, achieving an AUC of 0.871 in a combined validation cohort. They also used SHAP analysis to determine the relative contribution of individual variables to model predictions, emphasizing the importance of interpretability in ML-based FA assessment. Although their study addressed a different clinical outcome, it supports the potential role of ML approaches in pediatric FA evaluation. Similarly, in our study, SHAP analysis improved the interpretability of the evaluated prediction models by identifying disease severity and early disease onset as major contributors to FA prediction.

ML approaches are increasingly used in clinical prediction because they can evaluate multiple interrelated variables simultaneously and identify complex patterns within clinical data [23]. Among tree-based gradient boosting algorithms, XGBoost and CatBoost are particularly suited to capturing potentially nonlinear relationships among predictors [9,24,25]. An important finding of the present study is that different ML algorithms demonstrated distinct performance profiles despite being trained on the same dataset. CatBoost provided the most balanced diagnostic performance, combining high sensitivity (0.85) and specificity (0.91) with the highest AUC (0.91). In contrast, XGBoost achieved the highest specificity (0.96) after incorporation of laboratory variables, suggesting a stronger ability to correctly identify children without FA; however, its sensitivity remained lower (0.70) than that of the CatBoost clinical model. LightGBM using clinical features achieved the same sensitivity as CatBoost (0.85), but its specificity was lower (0.82), whereas logistic regression showed more limited sensitivity with both clinical features alone (0.65) and combined clinical-laboratory features (0.55).

These findings indicate that algorithm selection should not be based solely on AUC values, but also on the intended clinical application of the model. From a clinical perspective, sensitivity and specificity may be as important as overall discrimination. In young children, failure to identify FA risk may delay further diagnostic evaluation and prolong exposure to potentially relevant allergens, whereas low specificity may lead to unnecessary elimination diets, nutritional concerns, and additional diagnostic procedures. In this context, CatBoost achieved a favorable balance between these competing objectives, supporting its potential utility as a clinical risk-stratification tool. This distinction is clinically relevant because ML-based approaches can use routinely collected data while also allowing the incremental value of additional diagnostic variables to be assessed [22]. In our study, the inclusion of the evaluated laboratory variables (absolute eosinophil count, eosinophil percentage, and total IgE level) did not substantially improve overall model performance. These variables were intentionally selected to determine whether FA risk could be predicted using routinely available clinical characteristics and basic laboratory parameters without relying on allergen-specific diagnostic tests. This finding should not be generalized to other potentially informative biomarkers, such as food-specific IgE levels, skin prick test wheal sizes, or eosinophil-derived biomarkers, which were not included in the prediction models. In the CatBoost model, the addition of these laboratory variables did not increase the AUC and was associated with reduced sensitivity. These findings suggest that routinely available clinical characteristics may already capture much of the predictive information required for FA risk assessment in young children with AD.

Taken together, our findings suggest that a prediction model based on readily available clinical characteristics may provide useful guidance in the evaluation of FA risk among young children with AD. In particular, the strong performance of CatBoost using clinical features alone may enhance its applicability across different levels of pediatric care, including primary care, general pediatrics, and pediatric allergy practice. Such a model may help identify children who warrant further diagnostic evaluation for FA; however, it should be considered a clinical risk-stratification tool rather than an independent diagnostic method.

Beyond its primary findings, several aspects of this study contribute to the literature. First, it specifically focused on children younger than 2 years with established AD, a population known to be at particularly high risk for FA. Second, multiple ML algorithms, including gradient boosting models, were systematically compared within the same cohort. Third, the study evaluated the incremental contribution of laboratory biomarkers beyond routine clinical variables. Finally, model interpretability was enhanced through SHAP-based feature analysis, allowing better understanding of the factors driving prediction.

This study has several limitations, including its retrospective design and the relatively limited sample size for prediction model development. In addition, although 113 patients were excluded because they did not meet the eligibility criteria or had incomplete data, the specific reasons for exclusion were not systematically recorded separately. Therefore, the exact number of patients excluded due to incomplete data could not be reported. Additionally, relatively wide 95% confidence intervals were observed for some model performance metrics. Although comparative performance metrics were reported, differences in model performance were not formally tested. Furthermore, the cohort represents a clinically relevant high-risk population of young children with AD, but the absence of external validation limits the generalizability of the present findings.

Another limitation relates to outcome ascertainment. FA was diagnosed according to established clinical criteria, but not all patients underwent OFC. In addition, the specific diagnostic pathway leading to the final diagnosis was not systematically recorded as a separate variable in the study database. Consequently, the proportion of patients diagnosed by OFC versus convincing clinical history with documented sensitization could not be reported, and the possibility of verification bias cannot be excluded. Furthermore, the laboratory feature set was intentionally restricted to routinely available basic laboratory variables—absolute eosinophil count, eosinophil percentage, and total IgE level—in accordance with the study objective. Therefore, the findings regarding the incremental value of laboratory variables should not be generalized to allergen-specific or other potentially informative biomarkers, such as food-specific IgE levels, SPT wheal sizes, or eosinophil-derived biomarkers. Moreover, the clinical presentation leading to the diagnosis of FA (immediate IgE-mediated symptoms versus AD exacerbation) was not systematically recorded as a separate variable. Consequently, these subgroups could not be analyzed separately or reported. Although the SCORAD value used in the prediction models was obtained at the initial presentation, some conceptual overlap between baseline AD severity and the outcome definition cannot be completely excluded because AD exacerbation contributed to the diagnosis of FA in some patients. Nevertheless, the strong performance observed on the holdout test dataset supports further evaluation of this approach in larger, prospective, multicenter cohorts.

## 5. Conclusions

Among the evaluated models, CatBoost demonstrated the best overall performance for predicting FA in children aged <2 years with AD, achieving strong discriminatory ability using clinical features alone (AUC, 0.91). A model based on readily available clinical information may support the identification of young children with AD who warrant further diagnostic evaluation for FA across different levels of pediatric care. Prospective external validation is required before its use in routine clinical practice.

## Figures and Tables

**Table 1:** Demographic and clinical characteristics of children with atopic dermatitis according to food allergy status.

Characteristic	Total (*n* = 435)	Food Allergy (+) (*n* = 101)	Food Allergy (−) (*n* = 334)	*p* Value
Age at presentation, months, median (IQR)	7.4 (4.8–10.8)	6.1 (4.1–8.6)	7.9 (5.2–11.3)	<0.001
Age at onset of AD, months, median (IQR)	4.0 (2.0–6.0)	2.0 (2.0–3.0)	4.0 (3.0–6.0)	<0.001
Age at diagnosis of AD, months, median (IQR)	5.0 (4.0–8.0)	4.0 (3.0–5.0)	6.0 (4.0–9.0)	<0.001
Duration of AD, months, median (IQR)	2.0 (1.0–4.0)	3.0 (2.0–5.0)	2.0 (1.0–4.0)	0.005
Female, n (%)	185 (42.5)	37 (36.6)	148 (44.3)	0.171
Preterm birth, n (%)	18 (4.1)	3 (3.0)	15 (4.5)	0.775
Exclusive breastfeeding, n (%)	241 (55.4)	47 (46.5)	194 (58.1)	0.041
Family history of atopy, n (%)	80 (18.4)	28 (27.7)	52 (15.6)	0.006
Parental consanguinity, n (%)	32 (7.4)	9 (8.9)	23 (6.9)	0.495
SCORAD index, n (%)				<0.001
Mild, <25	345 (79.3)	41 (40.6)	304 (91.0)	
Moderate, 25–50	88 (20.2)	58 (57.4)	30 (9.0)	
Severe, ≥50	2 (0.5)	2 (2.0)	0	
Sites of Eczema scores, n (%)				<0.001
1	268 (61.6)	18 (17.8)	250 (74.9)	
2	116 (26.7)	43 (42.6)	73 (21.9)	
3	37 (8.5)	27 (26.7)	10 (3.0)	
4	14 (3.2)	13 (12.9)	1 (0.3)	

Abbreviations: AD, atopic dermatitis; IQR, interquartile range; SCORAD, SCORing Atopic Dermatitis.

**Table 2:** Performance of machine learning models for predicting food allergy.

Machine Learning Models	Features	AUC (95% CI)	Sensitivity (95% CI)	Specificity (95% CI)	Accuracy (95% CI)	Precision (95% CI)	F1-Score (95% CI)
CatBoost	Clinical features	0.91 (0.84–0.97)	0.85 (0.67–1.00)	0.91 (0.83–0.97)	0.90 (0.83–0.96)	0.74 (0.54–0.92)	0.79 (0.63–0.90)
CatBoost	Clinical and laboratory features	0.91 (0.84–0.97)	0.60 (0.38–0.82)	0.91 (0.84–0.97)	0.84 (0.76–0.91)	0.67 (0.44–0.89)	0.63 (0.43–0.79)
XGBoost	Clinical features	0.85 (0.76–0.93)	0.65 (0.44–0.86)	0.88 (0.80–0.95)	0.83 (0.75–0.90)	0.62 (0.40–0.83)	0.63 (0.44–0.78)
XGBoost	Clinical and laboratory features	0.88 (0.79–0.95)	0.70 (0.50–0.90)	0.96 (0.90–1.00)	0.90 (0.83–0.95)	0.82 (0.64–1.00)	0.76 (0.58–0.90)
LightGBM	Clinical features	0.89 (0.82–0.96)	0.85 (0.68–1.00)	0.82 (0.72–0.90)	0.83 (0.75–0.90)	0.59 (0.40–0.77)	0.69 (0.52–0.83)
LightGBM	Clinical and laboratory features	0.87 (0.78–0.94)	0.75 (0.56–0.92)	0.87 (0.78–0.94)	0.84 (0.76–0.91)	0.63 (0.42–0.81)	0.68 (0.50–0.82)
Logistic regression	Clinical features	0.86 (0.75–0.95)	0.65 (0.42–0.85)	0.88 (0.79–0.95)	0.83 (0.75–0.90)	0.62 (0.40–0.82)	0.63 (0.43–0.79)
Logistic regression	Clinical and laboratory features	0.86 (0.75–0.95)	0.55 (0.33–0.75)	0.93 (0.86–0.98)	0.84 (0.75–0.91)	0.69 (0.43–0.91)	0.61 (0.39–0.77)

Abbreviations: AD, atopic dermatitis; AUC, area under the curve; CatBoost, categorical boosting; CI, confidence interval; LightGBM, Light Gradient Boosting Machine; XGBoost, Extreme Gradient Boosting. Clinical features included age at presentation, age at onset of AD, age at diagnosis of AD, duration of AD, sex, gestational age, infant feeding pattern, family history of atopy, parental consanguinity, Sites of Eczema scores, and SCORAD index. Laboratory features included absolute eosinophil count, eosinophil percentage, and total IgE level.

## Data Availability

The data presented in this study are available on request from the corresponding author. The data are not publicly available due to privacy or ethical restrictions.

## Abbreviations

The following abbreviations are used in this manuscript:

AD	Atopic dermatitis
FA	Food allergy
AI	Artificial intelligence
ML	Machine learning
SCORAD	Scoring Atopic Dermatitis
SoE	Sites of Eczema
SPT	Skin prick testing
sIgE	Specific IgE
OFC	Oral food challenge
SHAP	SHapley Additive exPlanations
IQR	Interquartile range
AUC	Area under the curve
ROC	Receiver operating characteristic
